# Supplementary figures and images for: Protein sequence editing defines distinct and overlapping functions of SKN-1A/Nrf1 and SKN-1C/Nrf2
Source: PLoS Genet. 2025 Jul 7;21(7):e1011780. doi: 10.1371/journal.pgen.1011780 (PMC12251208; doi:10.1371/journal.pgen.1011780)

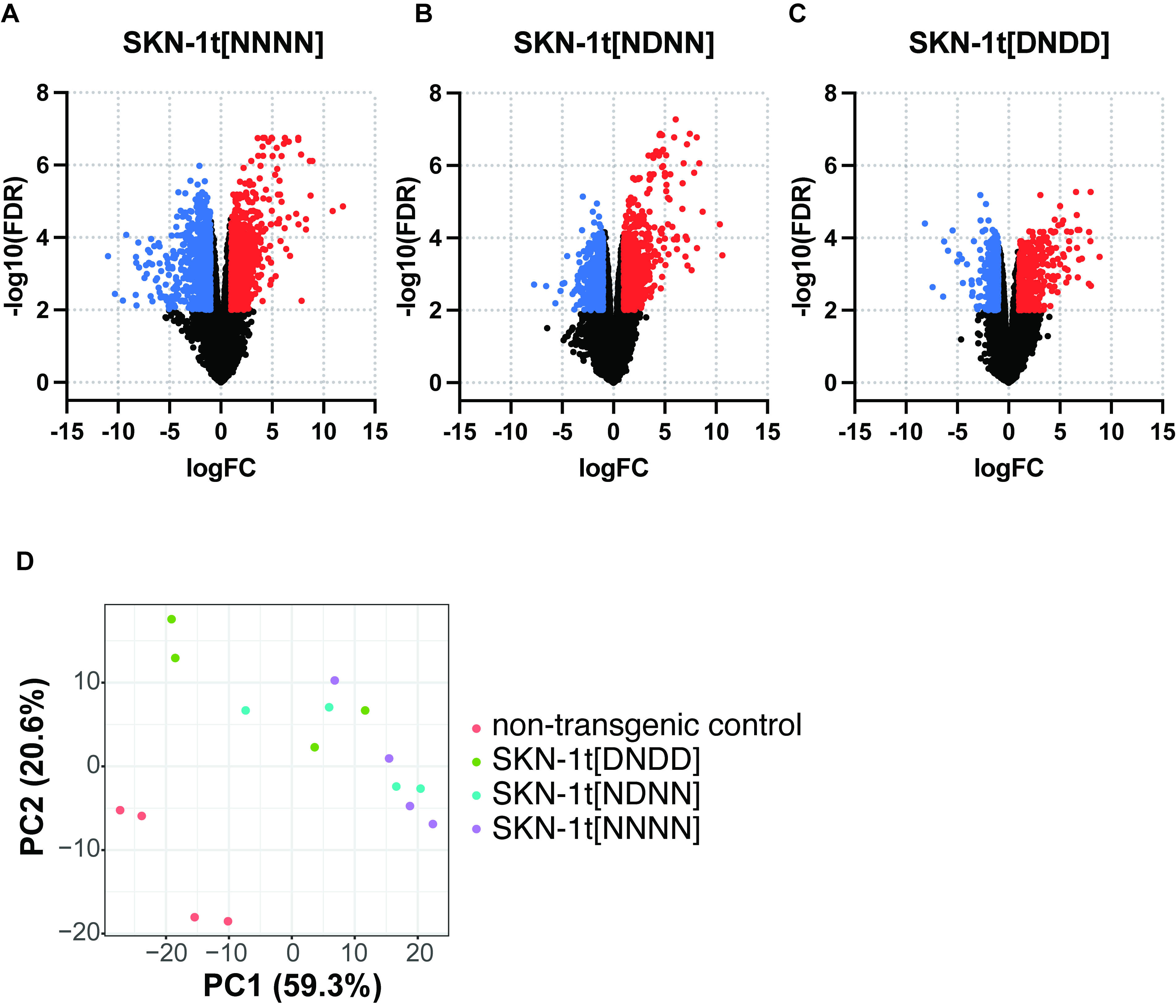

Supplement: S1 Fig — A-C) Volcano plots showing differential gene expression in SKN-1t transgenic strains compared to the non-transgenic wild-type control. Significantly upregulated and downregulated genes (fold change >2, FDR < 0.01) are indicated in red and in blue, respectively. Genes that do not show significant differential expression are indicated in black. D) Principal Component Analysis comparison of gene expression profiles showing non-transgenic control and SKN-1t transgenic samples. (TIF) [file pgen.1011780.s001.tif]

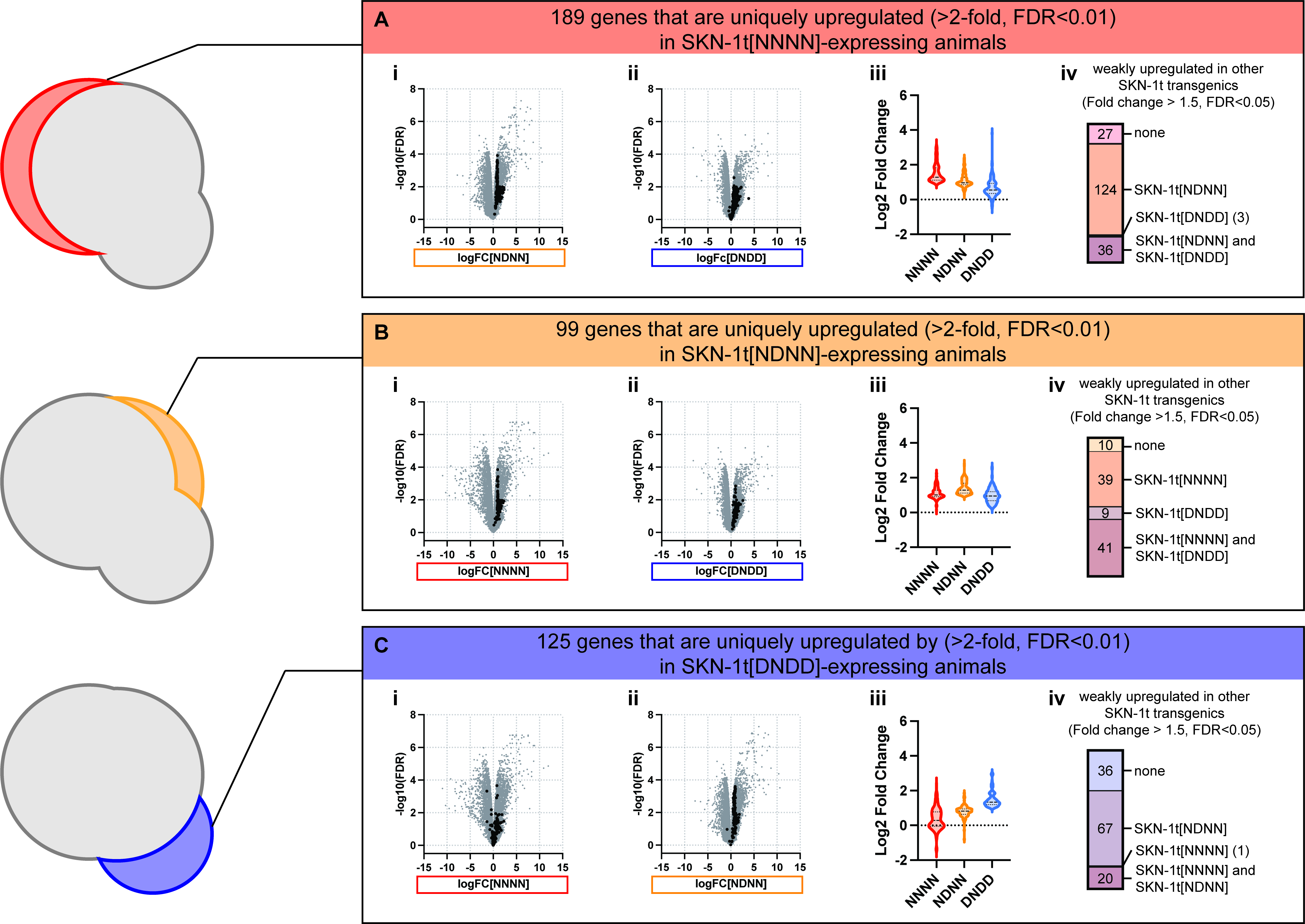

Supplement: S2 Fig — A) Analysis of 189 genes that appear to be uniquely upregulated in SKN-1t[NNNN] transgenic animals (>2-fold, FDR < 0.01). (i, ii) Volcano plots showing differential expression of uniquely SKN-1t[NNNN]-upregulated genes in SKN-1t[NDNN] (i) and SKN-1t[DNDD] (ii) transgenic animals. In each graph, the 189 genes are indicated in black, all other genes are indicated in gray. (iii) Violin plot comparing log2 fold change of the 189 uniquely SKN-1t[NNNN]-upregulated genes in each SKN-1t transgenic strain. These genes are skewed towards upregulation in SKN-1t[NDNN] and SKN-1t[DNDD] transgenic animals. (iv) Proportion of the 189 uniquely SKN-1t[NNNN]-upregulated genes that are upregulated in SKN-1t[NDNN] and/or SKN-1t[DNDD] at a lower stringency cutoff (>1.5-fold, FDR < 0.05). Most genes are upregulated in SKN-1t[NDNN] transgenics, and a smaller fraction are also upregulated in SKN-1t[DNDD] transgenics at this cutoff. B) Analysis of 99 genes that appear to be uniquely upregulated in SKN-1t[NDNN] transgenic animals (>2-fold, FDR < 0.01). (i, ii) Volcano plots showing differential expression of uniquely SKN-1t[NDNN]-upregulated genes in SKN-1t[NNNN] (i) and SKN-1t[DNDD] (ii) transgenic animals. In each graph, the 99 genes are indicated in black, all other genes are indicated in gray. (iii) Violin plot comparing log2 fold change of the 99 uniquely SKN-1t[NDNN]-upregulated genes in each SKN-1t transgenic strain. These genes are skewed towards upregulation in SKN-1t[NNNN] and SKN-1t[DNDD] transgenic animals. (iv) Proportion of the 99 uniquely SKN-1t[NNNN]-upregulated genes that are upregulated in SKN-1t[NDNN] and/or SKN-1t[DNDD] at a lower stringency cutoff (>1.5-fold, FDR < 0.05). Most genes are upregulated in at least one of the two other transgenic strains at this cutoff. C) Analysis of 125 genes that appear to be uniquely upregulated in SKN-1t[DNDD] transgenic animals (>2-fold, FDR < 0.01). (i, ii) Volcano plots showing differential expression of uniquely SKN-1t[DNDD]-upregula [file pgen.1011780.s002.tif]

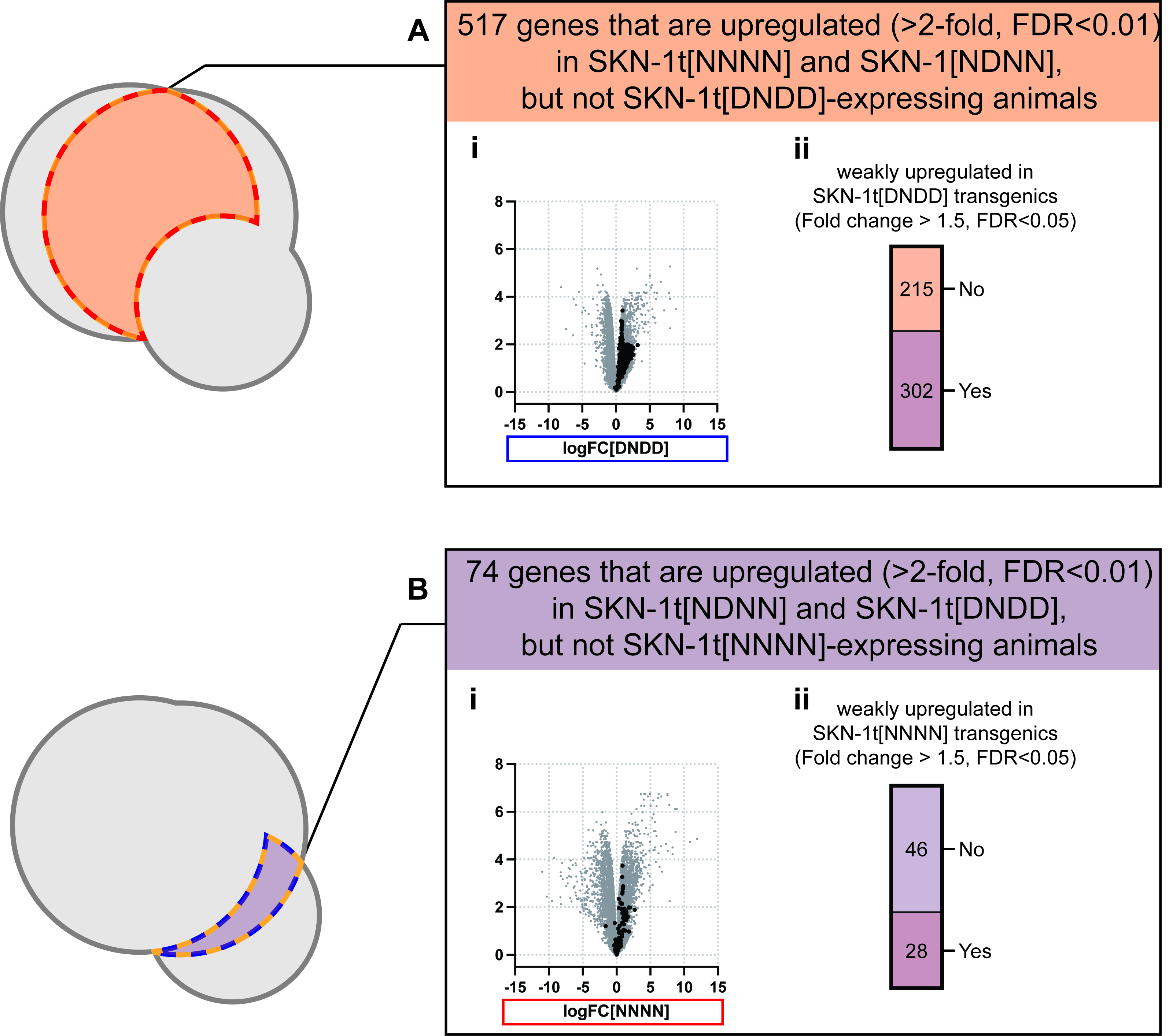

Supplement: S3 Fig — A) Analysis of 517 genes that appear to be upregulated in SKN-1t[NNNN] and SKN-1-NDNN] transgenic animals (>2-fold, FDR < 0.01), but not in SKN-1t[DNDD] transgenic animals. (i) Volcano plots showing differential expression of these 517 genes SKN-1t[DNDD] transgenic animals. The 517 genes are indicated in black; all other genes are indicated in gray. (ii) Proportion of these 517 genes that are upregulated in SKN-1t[DNDD] transgenics at a lower stringency cutoff (>1.5-fold, FDR < 0.05). Most genes are upregulated in SKN-1t[DNDD] transgenics at this cutoff. B) Analysis of 74 genes that appear to be upregulated in SKN-1t[NDNN] and SKN-1-DNDD] transgenic animals (>2-fold, FDR < 0.01), but not in SKN-1t[NNNN] transgenic animals. (i) Volcano plots showing differential expression of these 74 genes SKN-1t[NNNN] transgenic animals. The 74 genes are indicated in black; all other genes are indicated in gray. (ii) Proportion of these 75 genes that are upregulated in SKN-1t[NNNN] transgenics at a lower stringency cutoff (>1.5-fold, FDR < 0.05). Approximately one third of the 74 genes are upregulated in SKN-1t[NNNN] transgenics at this cutoff. (TIF) [file pgen.1011780.s003.tif]

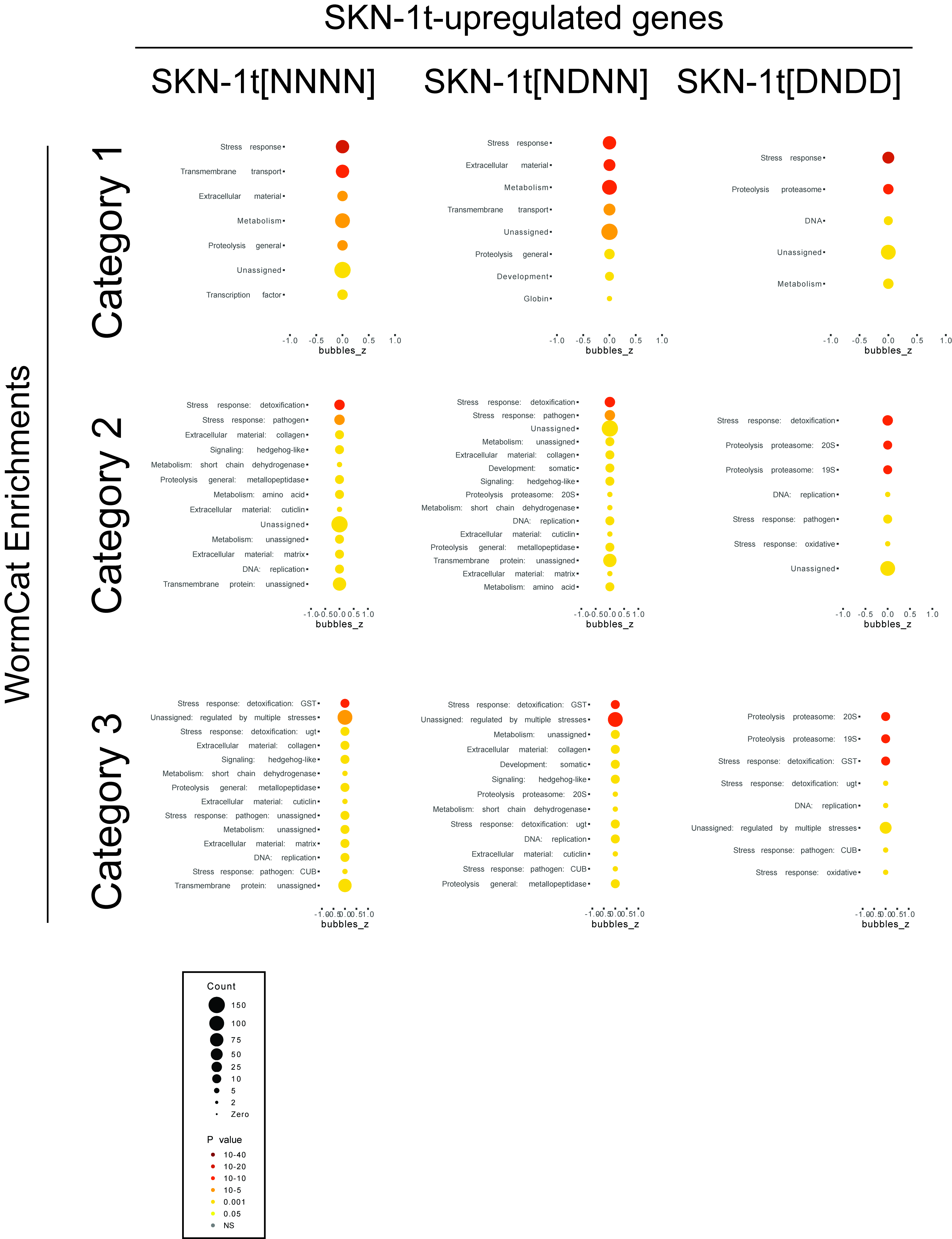

Supplement: S4 Fig — Functional enrichments of genes upregulated in each SKN-1t transgenic strain (see Fig 1A and 1B), as assessed by WormCat. (TIF) [file pgen.1011780.s004.tif]

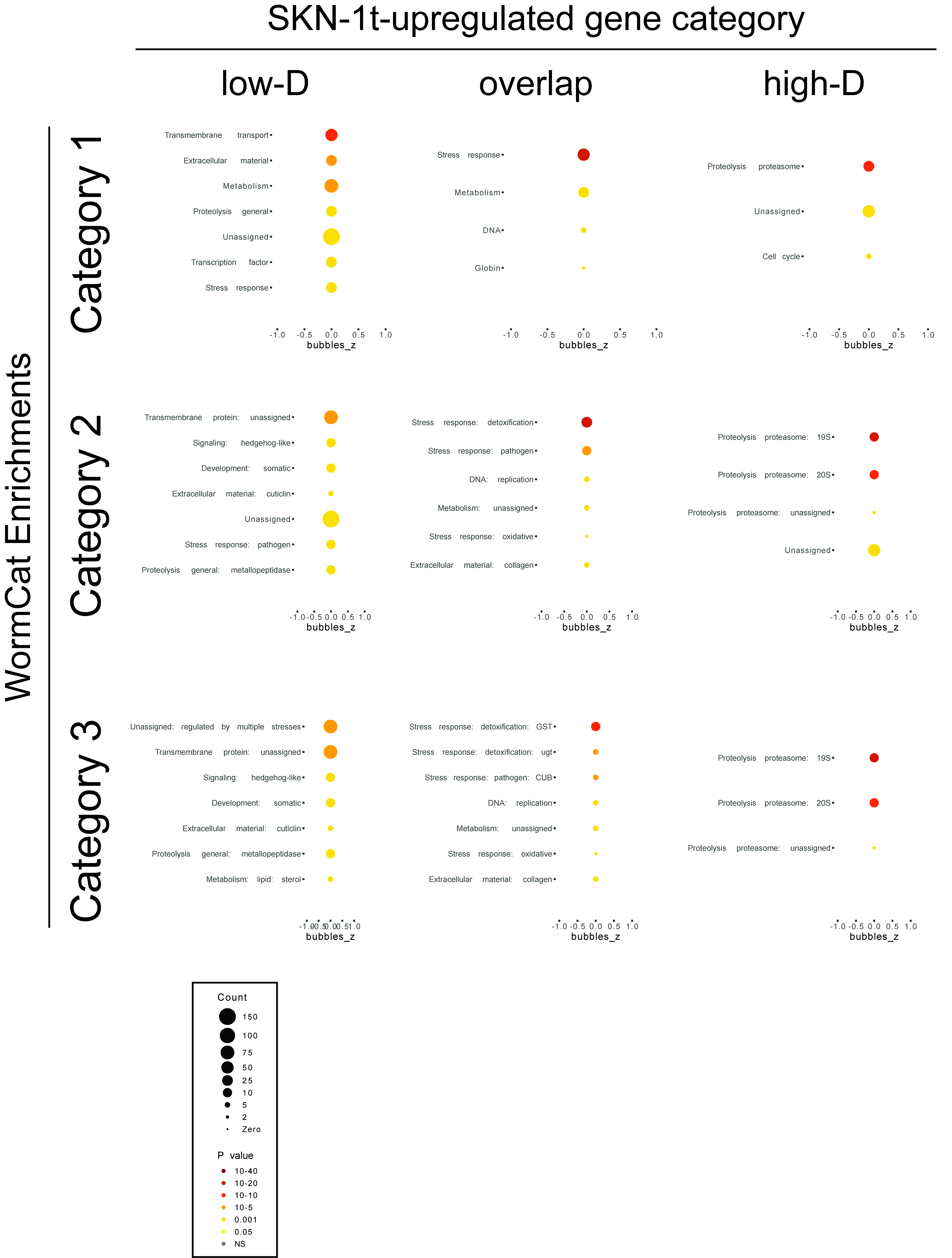

Supplement: S5 Fig — Functional enrichments of SKN-1t-upregulated genes categories that are differentially affected by sequence editing mutations, as assessed by WormCat. The three classes are defined as follows (see Fig 2A for more detail): (1) ‘high-D’ genes are upregulated (>2-fold, FDR < 0.01) in SKN-1t[DNDD], but not in SKN-1t[NNNN] transgenics; (2) ‘overlap’ genes are upregulated (>2-fold, FDR < 0.01) in SKN-1t[NNNN], SKN-1t[NDNN] and SKN-1t[DNDD] transgenics; (3) ‘low-D’ genes are upregulated in SKN-1t[NNNN] and/or SKN-1t[NDNN], but not in SKN-1t[DNDD] transgenics. (TIF) [file pgen.1011780.s005.tif]

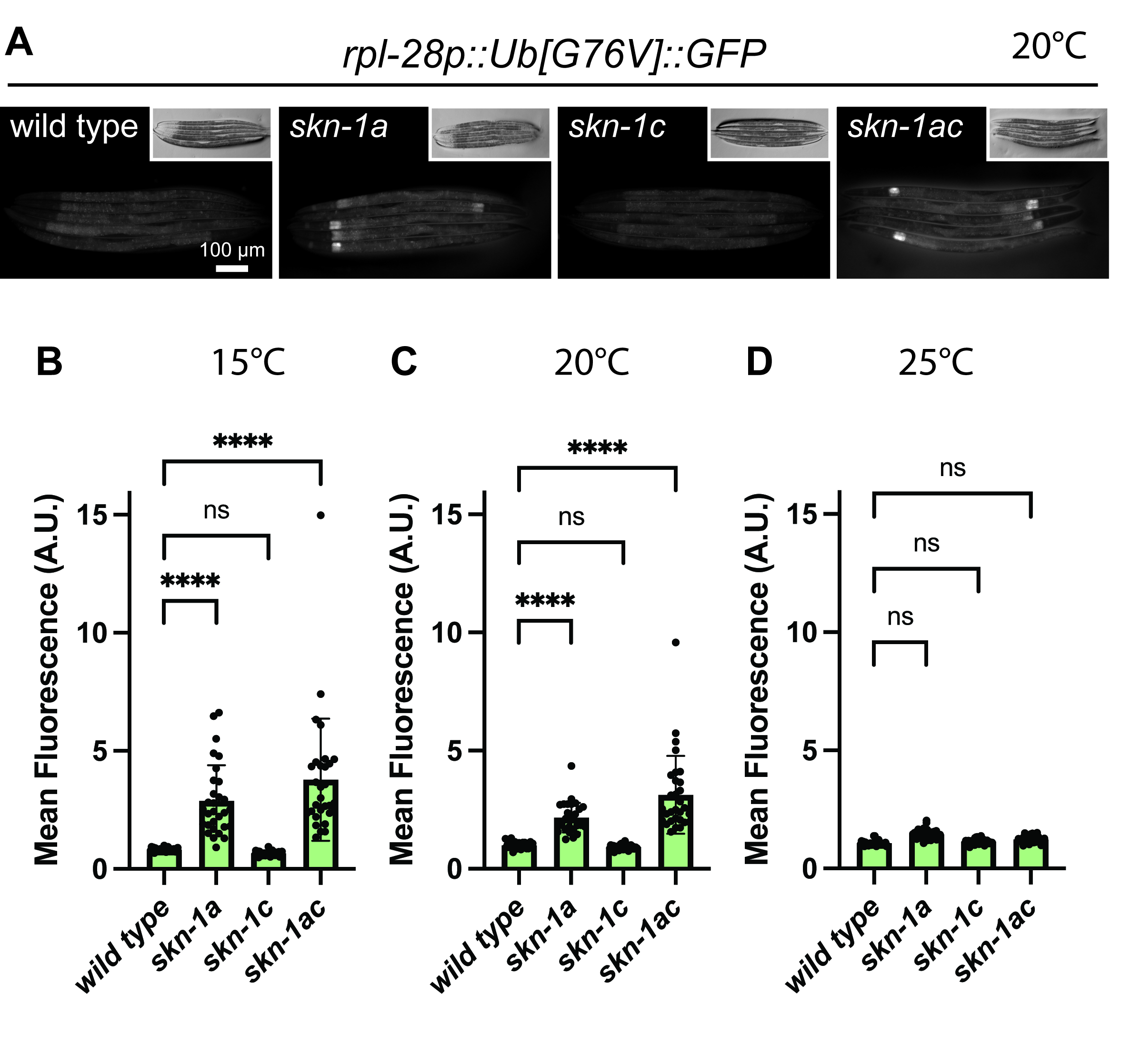

Supplement: S6 Fig — A) Fluorescence images showing accumulation of Ub[G76V]::GFP in skn-1 mutants at the L4 stage. Increased accumulation is observed in skn-1a and skn-1ac mutants, but not in skn-1c mutants. Scale bar shows 100 μm. B) Quantification of Ub[G76V]::GFP in skn-1 mutants raised at different temperatures to the L4 stage. At 15°C and 20°C, increased Ub[G76V]::GFP is detected in skn-1a and skn-1ac mutants, but not skn-1c mutant animals. In animals raised at 25°C, there are no detectable changes in any of the mutants. n = 30 animals were imaged for each genotype at each temperature. Error bars show mean ± SD. **** p < 0.0001, *** p < 0.001, ordinary two-way ANOVA with Tukey’s multiple comparisons test. Numerical data for panels B-D are available in S1 Data. (TIF) [file pgen.1011780.s006.tif]

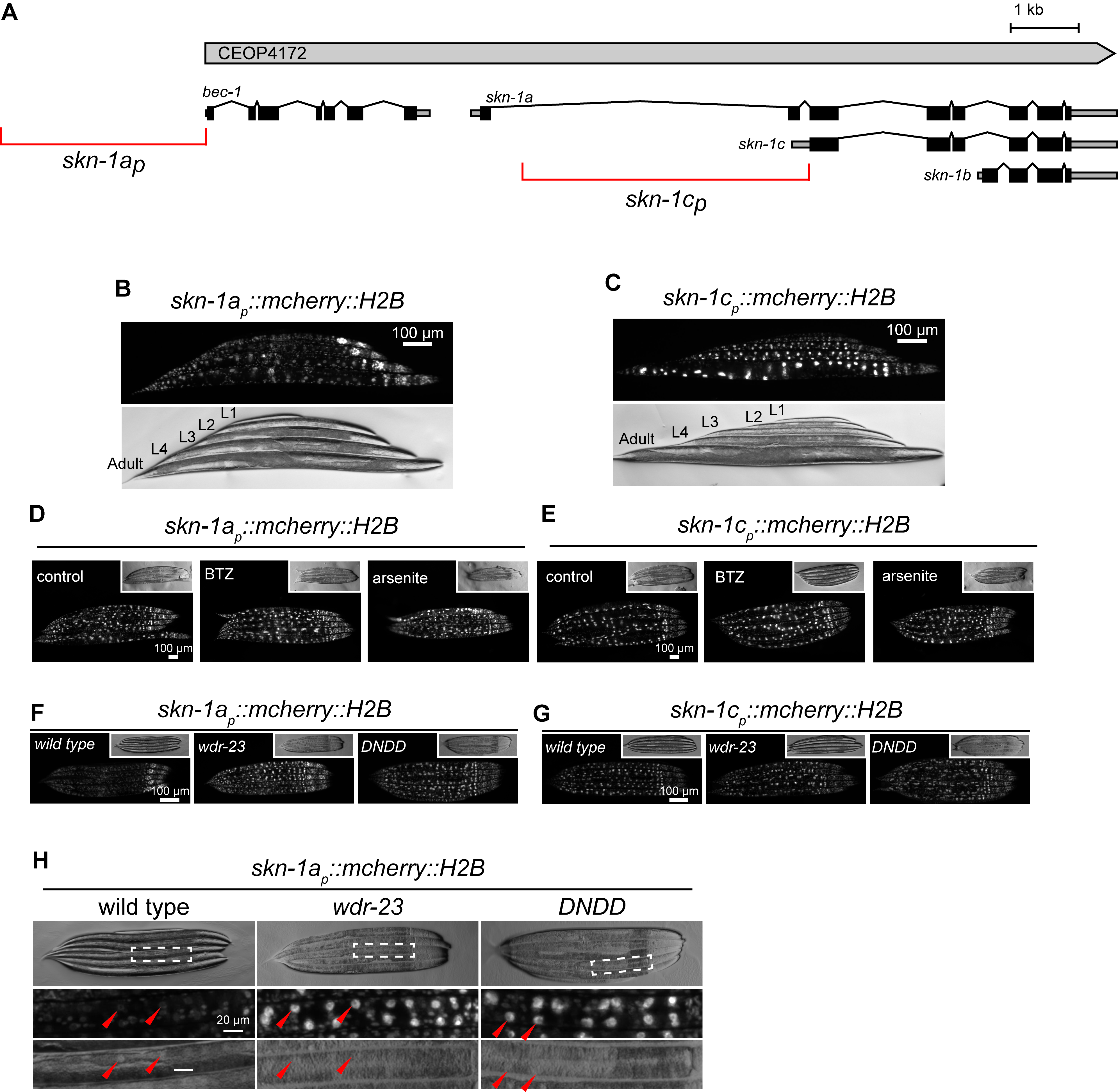

Supplement: S7 Fig — A) Schematic showing CEOP4172 including the bec-1 and skn-1 loci. The DNA fragments corresponding to the promoter of skn-1a/COEP4172 (skn-1ap) and the promoter of skn-1c (skn-1cp) are shown. B) Fluorescence image showing expression of mCherry::H2B under the skn-1a/CEOP4172 promoter at each stage of larval development and in adults. The reporter is expressed at all stages and in most cells. The most prominent expression is detected in unidentified cells in the head. Scale bar shows 100 μm. C) Fluorescence image showing expression of mCherry::H2B under the skn-1c promoter at each stage of larval development and in adults. The reporter is expressed at all stages in most cells. The most prominent expression is detected in the intestine. Scale bar shows 100 μm. D, E) Fluorescence micrographs showing skn-1ap::mCherry::H2B (d) and skn-1cp::mCherry::H2B (e) expression in animals exposed to the proteasome inhibitor bortezomib (BTZ, 0.4 µg/ml (1.04 µM)) or Arsenite (3 mM) compared to untreated controls. In each case, expression of the reporter is unchanged under stress conditions. Scale bar shows 100 μm. F, G) Fluorescence micrographs showing skn-1ap::mCherry::H2B (F) and skn-1cp::mCherry::H2B (G) expression in wdr-23(tm1817) mutants and in SKN-1t[DNDD] transgenics compared to wild type controls. Images show L4 stage animals. The expression of the skn-1c reporter is unchanged, whereas the expression of the skn-1a reporter is increased in intestinal cells. Scale bar shows 100 μm. H) Fluorescence micrographs showing increased expression of skn-1ap::mCherry::H2B in intestinal cells of wdr-23(tm1817) mutant animals and SKN-1t[DNDD] transgenic animals compared to wild type controls. Images show L4 stage animals. Scale bar shows 20 μm. (TIF) [file pgen.1011780.s007.tif]

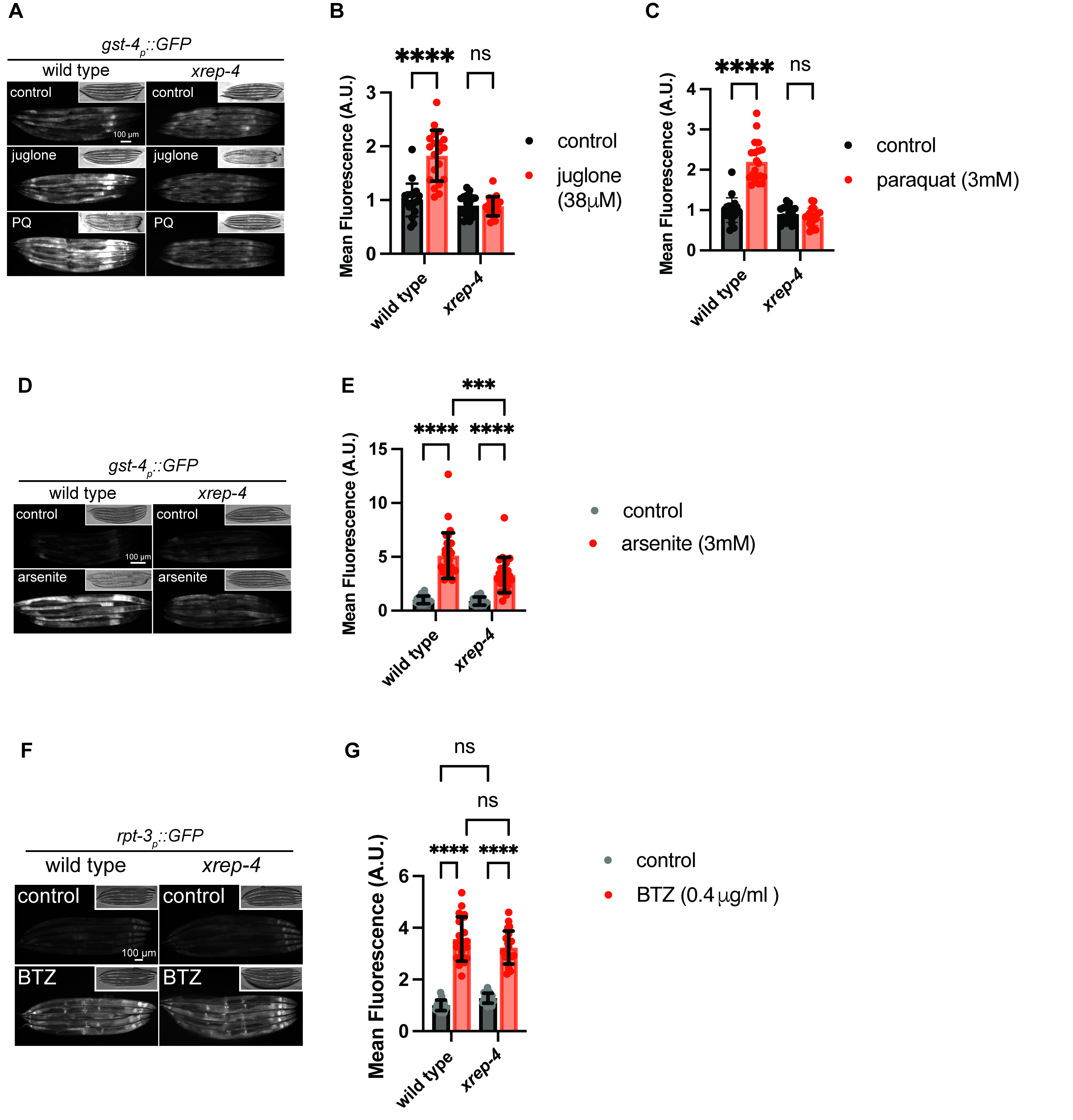

Supplement: S8 Fig — A) Fluorescence micrographs showing gst-4p::gfp expression in animals exposed to juglone (38 μΜ, 4 hours) or paraquat (3 mM, 4 hours). The reporter is induced in the wild type but not in xrep-4 mutant animals. Scale bar shows 100 μm. B, C) Quantification of gst-4p::gfp induction in animals exposed to juglone (38 μΜ, 4 hours) or paraquat (3 mM, 4 hours), as shown in (A). Error bars show mean ± SD. **** p < 0.0001, ns p > 0.05, ordinary two-way ANOVA with Sidak’s multiple comparisons test. D) Fluorescence micrographs showing gst-4p::gfp expression in animals exposed to arsenite (3 mM, 4 hours). Induction of the reporter is attenuated in xrep-4 mutant animals. Scale bar shows 100 μm. E) Quantification of gst-4p::gfp expression in animals exposed to arsenite (3 mM, 4 hours), as shown in (D). Error bars show mean ± SD. **** p < 0.0001, ordinary two-way ANOVA with Sidak’s multiple comparisons test. F) Fluorescence micrographs showing rpt-3p::gfp expression in animals exposed to bortezomib (BTZ, 0.4 μg/ml, approximately 20 hours). Induction is not altered in xrep-4 mutants compared to the wild type. Scale bar shows 100 μm. G) Quantification of rpt-3p::gfp expression in animals exposed to bortezomib (BTZ, 0.4 μg/ml), as shown in (F). Error bars show mean ± SD. **** p < 0.0001, ordinary two-way ANOVA with Sidak’s multiple comparisons test. Numerical data for panels B, C, E, and G are available in S1 Data. (TIF) [file pgen.1011780.s008.tif]
